# Personality, Psychopathology, and Psychotherapy: A Pre-specified Analysis Protocol for Confirmatory Research on Personality–Psychopathology Associations in Psychotherapy Outpatients

**DOI:** 10.3389/fpsyt.2017.00009

**Published:** 2017-02-01

**Authors:** Michael P. Hengartner, Misa Yamanaka-Altenstein

**Affiliations:** ^1^Department of Applied Psychology, Zurich University of Applied Sciences, Zurich, Switzerland; ^2^Klaus-Grawe-Institute for Psychological Therapy, Zurich, Switzerland

**Keywords:** psychotherapy, psychopathology, personality, confirmatory research, psychiatric treatment, study protocol

## Abstract

The role of personality trait variation in psychopathology and its influence on the outcome of psychotherapy is a burgeoning field. However, thus far most findings were based on controlled clinical trials that may only poorly represent real-world clinical settings due to highly selective samples mostly restricted to patients with major depression undergoing antidepressive medication. Focusing on personality and psychopathology in a representative naturalistic sample of psychotherapy patients is therefore worthwhile. Moreover, up to date hardly any confirmatory research has been conducted in this field. Strictly confirmatory research implies two major requirements: firstly, specific hypotheses, including expected effect sizes and statistical approaches to data analysis, must be detailed prior to inspection of the data, and secondly, corresponding protocols have to be published online and freely available. Here, we introduce a longitudinal naturalistic study aimed at examining, firstly, the prospective impact of baseline personality traits on the outcome of psychotherapy over a 6-month observation period; secondly, the stability and change in personality traits over time; thirdly, the association between longitudinal change in psychopathology and personality; fourthly, the agreement between self-reports and informant rating of personality; and fifthly, the predictive validity of personality self-reports compared to corresponding informant ratings. For it, we comprehensively state *a priori* hypotheses, predict the expected effect sizes and detail the statistical analyses that we intend to conduct to test these predictions. Such a stringent confirmatory design increases the transparency and objectivity of psychopathological research, which is necessary to reduce the rate of false-positive findings and to increase the yield of scientific research.

## Introduction

### The Importance of Personality for Public Mental Health and Psychotherapy

The global burden of disease attributable to mental disorders is tremendous (1), in particular in high-income Western societies, including Europe and the United States of America (2, 3). Targeting modifiable risk factors for severe psychopathology has therefore highest priority. Previous research has confirmed that maladaptive personality is a crucial prospective risk factor for ill-health, all-cause mortality, and social functioning deficits (4–7). Meta-analyses have further revealed that personality shows a substantial association with subjective well-being (8) and relationship satisfaction (9). Interestingly, behavioral genetics suggest that the pleiotropic genetic influences underlying personality trait variation fully account for the genetic interindividual differences in subjective well-being (10). Evidence from cross-sectional studies suggests that personality traits, in particular high neuroticism and low conscientiousness, strongly relate to psychopathology (11, 12). In longitudinal studies adjusted for baseline impairment, the prospective effect of personality traits on subsequent occurrence of psychopathology is considerably weaker but remains substantial (13–16). Moreover, evidence from several quantitative genetic studies suggests that neuroticism and internalizing disorders (i.e., depression, anxiety, and stress-related disorders) share approximately 50% of common genetic variance (17–19). Recent evidence has further demonstrated that neuroticism accounts for cognitive traits such as rumination and evaluation, which negatively impact on psychopathology (20, 21). In an attempt to quantify the economic costs attributable to both neuroticism and common mental disorders (i.e., depression, anxiety, and substance-use disorders) in the general population, Cuijpers and colleagues (22) found that the per capita excess costs for the 25% highest neuroticism scorers were approximately 2.5 time higher than the costs attributable to all common mental disorders combined. Accordingly, personality traits are considered among the most important modifiable factors influencing public health and psychiatric practice (4, 23–25). Because of its pervasive and persistent impact on (mental) health and functioning, personality necessarily needs to be considered in the planning and conduct of psychotherapy (26, 27). Indeed, original studies revealed that baseline personality significantly interferes with the course of psychopathology and the efficacy of psychotherapeutic interventions (28–30). It was further demonstrated that personality difficulties take considerably longer to treat than the common number of 15–19 sessions routinely administered in randomised controlled trials (RCT) (31, 32). In accordance, comorbid personality disorders, which can be modeled as maladaptive variants of normal personality traits (33–35), have shown to significantly reduce the treatment response in patients with mood and anxiety disorders (36–38) and to predict long-term functioning deficits and impairments (39–42). Taken together, these findings stress the importance of personality for public mental health and suggest that psychotherapy, which is the second most common treatment for mental disorders after psychotropic medication in Europe and the United States (43, 44), needs to carefully incorporate personality traits.

### What This Project Adds to the Literature

There are various gaps in the literature that need to be addressed to foster and enhance our understanding of personality effects on psychopathology over the course of psychotherapeutic interventions. So far most original studies on the influence of personality on the outcome of psychotherapy have been conducted as RCT under laboratory conditions and almost exclusively with depressed patients (28–30, 45, 46). Even though RCT are considered the gold standard to evaluate the efficacy of therapeutic interventions, they have various severe limitations, the most important being low external validity and poor generalizability (47, 48). That is, real-world effectiveness of psychotherapeutic (48) and pharmacological interventions (47) is often considerably lower than their efficacy under carefully controlled experimental conditions. There are at least two main reasons for that discrepancy. Firstly, highly selective inclusion criteria in RCT largely exclude participants with complicated comorbid disorders and personality pathology as well as patients who undergo additional treatments (48). Such a restrictive patient enrollment therefore produces biased samples with poor external validity that are not representative for the average patient seen in real-world clinical settings. Secondly, RCT are conducted strictly according to psychotherapy treatment manuals, while in real-world clinical settings therapists hardly consider and adhere to treatment manuals (49). It is therefore necessary to examine the association between personality and psychopathology in naturalistic real-world setting in order to generalize the findings from RCT. Because replication is a cornerstone of good psychological research (50–52), this is a timely objective. Moreover, almost all studies on the personality–psychopathology association conducted thus far relied exclusively on self-reports, even though self-reports and informant ratings of personality traits show only moderate agreement, in particular with respect to maladaptive neuroticism, pathological personality traits, and personality problems (53–55). Because both self- and other-reports of personality have incremental predictive validity above each other (56–58), the inclusion of an informant rating of personality functioning in the prediction of psychopathology and psychotherapy outcomes is thus worthwhile. Another important objective of the current project is to examine the stability of personality traits over the course of 6 months of psychotherapy using a longitudinal pre–post design. In community-based epidemiologic studies of personality–psychopathology associations, such a prospective design with baseline and follow-up assessment is an established standard protocol (16, 59–62), but with a few exceptions (61), these studies typically assess personality traits at baseline only. As for research in clinical samples, various longitudinal studies with pre–post assessment of personality traits have focused on the stability of personality traits in psychiatric patients (46, 63–65), but these chiefly tested the effects of antidepressive medication on personality in patients with major depression. There are, in addition, some studies focusing on the stability of personality in persons with substance-use problems (61, 66) or psychosis (67, 68), but none of these studies specifically examined outpatients undergoing psychotherapy. Because transdiagnostic psychological interventions targeted at modifying neuroticism have drawn broad interest in the research community (69, 70), focusing on the short-term stability of personality traits in unselected psychotherapy users with various diagnoses is thus worthwhile.

In sum, research has demonstrated that personality is crucial to psychopathology and public mental health (4, 71, 72). Even though some clinical psychologists have made compelling cases for the inclusion of personality in the planning and conduct of psychotherapy (26, 73, 74), the concept of personality remains largely underutilized in clinical settings (4, 27). For instance, before inception of this project, the psychotherapy institute serving as the study site that collects the data for the present project, did not apply any inventory of normal-range personality such as the five-factor model (FFM) of personality (75, 76), and this is certainly the rule rather than the exception in psychotherapy centers. Treatment protocols targeting neuroticism have been developed (69) and are now undergoing systematic evaluation (70). However, the treatment and the changeability of personality traits in psychotherapy, especially that of maladaptive personality, remain largely debated (46, 77, 78). The present research project thus aims to make several important contributions to the literature by applying a stringently objective and transparent confirmatory approach.

## Materials and Equipment

### Study Site and Sampling Procedure

All data will be collected at the Klaus-Grawe-Institute (KGI) for psychological therapy in Zurich, Switzerland, which is an outpatient psychotherapy center offering psychological therapy according to Grawe’s (79) framework of general psychology and psychotherapy. That approach aims at the comprehensive application of effective and evidence-based techniques of various psychotherapeutic orientations, including cognitive, behavioral, and interpersonal techniques. Every patient receives individually tailored treatment based on his/her individual biopsychosocial needs. As a result, each therapy is unique in its scope and application of specific psychotherapeutic techniques, which precludes the application of restrictive therapy manuals. Patients at the KGI present with heterogeneous mental health problems, including, but not limited to, depressive disorder, panic disorder, generalized anxiety disorder, post-traumatic stress disorder, adjustment disorder, acute stress disorder, burnout, substance-use disorder, eating disorder, and personality disorder. What makes this heterogeneous naturalistic sample so valuable is the fact that, in contrast to most RCT (48), inclusion is not restricted to specific psychiatric diagnoses. In fact, many clients treated at the KGI present with subclinical disorders and interpersonal problems and, accordingly, do not meet criteria for any diagnosis according to the International Classification of Disease 10th Edition (ICD-10) (80), which is in accord with the dimensional concept of mental disorders (81–83). In most clinical trials reviewed above, these patients would have been excluded, which restricts the external validity and generalizability of findings from such trials.

Collection of data will include all German-speaking patients between 18 and 65 years who started psychotherapy at the KGI in Zurich dating back to September 2015 when FFM measures of personality were included in the standard test battery. Even though therapy is also offered to English speaking patients, restriction to German-speaking patients was made because assessments in English comprise different questionnaires. Data from a comprehensive assessment at outset of the psychotherapy will be used as baseline measures. After 6 months, all patients will be reassessed with a subset of these measures unless their therapy ended prior to that. These data will be used as the 6-month follow-up assessment. Originally, we planned to reassess patients after 12 months (second follow-up), but we now came to realize that this is not feasible because there are not enough patients who remain incessantly in therapy for so long. The 12-month follow-up will therefore not be executed. Based on power analyses, we intend to proceed sampling until we have approximately *n* = 100 6-month follow-up assessments. Because sample size calculation is intricate and not readily applicable for generalised estimating equations (GEE) analysis, we relied on the following three sources: firstly, we scrutinized various sample size recommendations for correlated data published in the methodological literature (84–86); secondly, we tested different sample size estimations based on repeated measure ANOVA calculated with G*Power (87); and thirdly, we considered the significance levels for varying effect sizes as obtained from applications of GEE in our previous work (14, 88–90). The final sample size of *n* = 100 was then estimated based on all these sources for an expected moderate effect size (0.3 < *d* > 0.5), a significance level of α = 0.05 and power (1 − β) > 0.8.

### Outline of Confirmatory Designs

Psychological research is currently facing a substantial replication crisis (91), because its main focus lies on publishing spectacular positive findings instead of conducting methodologically sound replication studies (52, 92). As a result, many researchers involve in questionable research practices, meaning that data and statistical models are processed, transformed, and modified until one finds the desired association at *p* < 0.05 (93, 94). Such inadequate procedures, also referred to as *p*-hacking, often yield irreproducible, inflated, or false-positive associations (95–97). To avoid these systematic biases, various authors have called for more stringent confirmatory research designs (51, 98). To increase the objectivity and transparency of research designs, such an approach comprises that concise study and analysis protocols are published publicly online. These protocols not only define the exact study design, including sampling procedure, timing, and conduct of measurements, the applied assessment instruments, and the hypotheses to be tested but also which outcomes will be analyzed to test these predictions. To reduce the flexibility in analysis procedures, which markedly inflates the rate of false-positive findings (94), one therefore also prespecifies the exact statistical methods and how the outcome of interest will be modeled before the data are known. According to these state-of-the-art guidelines for confirmatory research (51, 98), we published the original study protocol submitted to the KEK publicly online using the Open Science Framework (https://osf.io/ukbs5/). The hypotheses related to the aims of this project stated in that document will be rephrased in more detail below.

### Instruments and Measures

Because this is a fully naturalistic study, we will use only instruments and measures that are routinely applied at the KGI to all patients. That is, to avoid methodological artifacts such as design or experimenter effects, we will not add additional assessment procedures that are not part of the basic psychotherapy evaluation. In consequence, the coverage and range of assessment instruments is limited those applied at the study site. However, note that there are further instruments applied at the KGI that are not considered in the present study, because they are administered only to selected patients with specific problems or because they were considered redundant for the present study aims. Normal-range personality will be assessed with a German adaptation (99) of the well-established Big Five Inventory (BFI) (100). The BFI is a brief self-report inventory capturing the basic structure of personality based on the broad domains of neuroticism, extraversion, openness, conscientiousness, and agreeableness. The BFI has been validated in diverse samples across nations, including English, German, Dutch, and Chinese, and is considered a reliable and valid short assessment of the FFM (99–102). Personality will be further assessed using the German adaptation (103) of the Inventory of Interpersonal Problems (IIP) (104). The IIP relies on the interpersonal circumplex model (105, 106) and captures personality difficulties based on the following eight primary domains of interpersonal problems, that is, domineering, vindictive, cold, socially avoidant, submissive, exploitable, overly nurturant, and intrusive. On a higher-order level, these domains collapse into the meta-factors of agency/dominance and communion/affiliation. The circumplex personality domains revealed strong and consistent associations with both the Big Five traits of extraversion and agreeableness and personality disorder dimensions (107–109), which is why personality pathology is considered a disorder of interpersonal behavior (110–112). In addition to the self-report IIP, an informant rating scale of the circumplex model will also be administered using the Impact Message Inventory-Circumplex (IMI-C) (113, 114). The informant rating will be provided by various sources; however, in most cases, the rater will be the partner/spouse or a family member. The BFI will be assessed at both baseline and 6-month follow-up, while IIP and IMI-C will be completed at baseline only due to their length.

**Figure 1:**
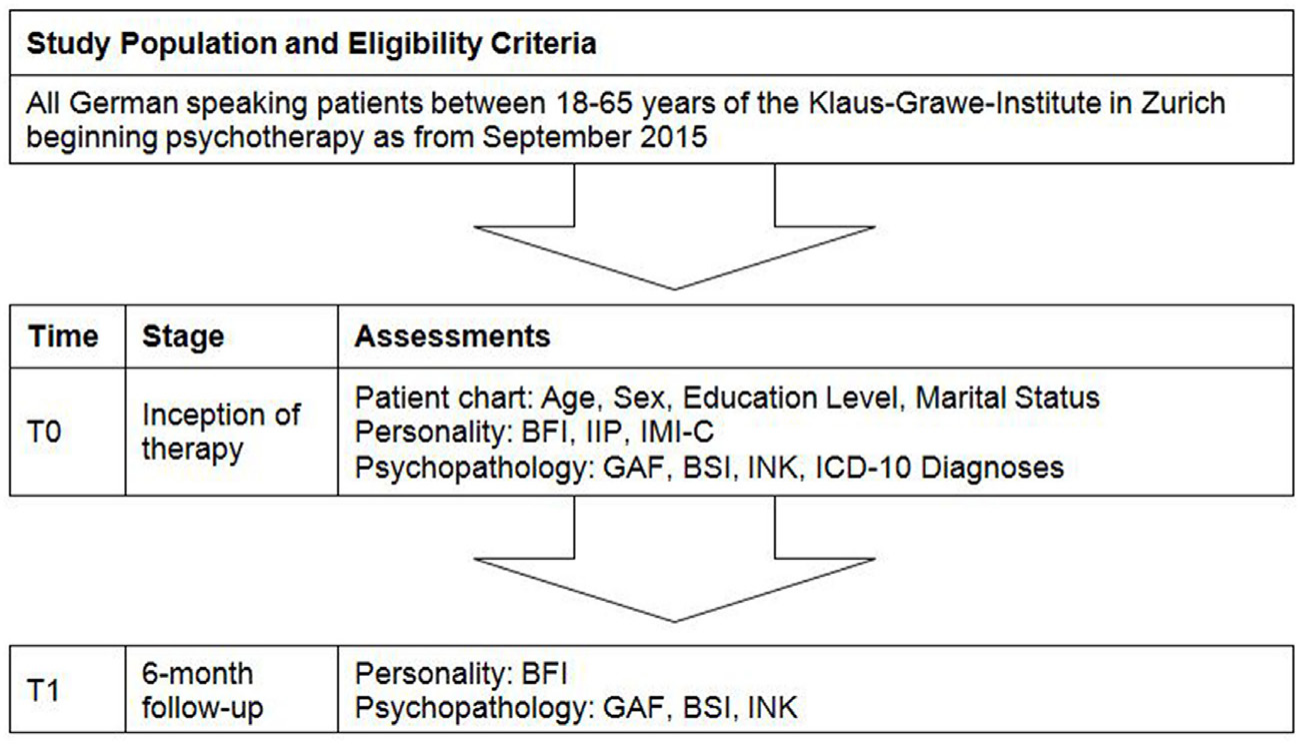
**Participant flowchart**. BFI, Big Five Inventory; IIP, Inventory of Interpersonal Problems; IMI-C, Impact Message Inventory-Circumplex; GAF, Global Assessment of Functioning; BSI, Brief Symptom Inventory; INK, Incongruence Questionnaire; ICD-10, International Classification of Diseases 10th Edition.

Subjective psychopathological symptoms will be assessed with the German translation (115) of the Brief Symptom Inventory (BSI) (116). The BSI measures psychopathology based on the following nine syndromes: somatization, obsessive–compulsive, interpersonal sensitivity, depression, anxiety, hostility, phobia, paranoia, and psychoticism. Although these scales demonstrated good reliability (115, 116), due to rather poor convergent and discriminant validity, it has been suggested to use only the total impairment score (117). As an alternative measure of subjective impairment we will use the incongruence questionnaire (INK) (118). The INK measures motivational incongruence, defined as the discrepancy between a person’s motivational goals and his/her perception of the actual fulfilment of his/her socio-affective needs. The INK has shown good psychometric properties and correlates strongly with psychopathological distress, which makes it a valuable tool for treatment evaluation in psychotherapy research and practice (118, 119). Finally, the treating psychotherapist will quantify the global functioning of the patient using the Global Assessment of Functioning (GAF) scale (120) in order to obtain an objective other rating of his/her psychopathological impairment. All measures of psychopathology, that is, BSI, INK, and GAF will be administered both at baseline and 6-month follow-up. In addition to the inventories and rating scales detailed above, information from the patient chart, including diagnoses according to ICD-10, age, sex, and education level, will also be recorded in the dataset. The flowchart is shown in Figure 1.

## Stepwise Procedures

This observational naturalistic study was approved by the Cantonal Ethics Committee of Zurich (KEK) in December 2015 (reference number 2015-0601). The project is financially supported by a grant from the OPO Foundation (reference number 2016-0038) awarded in June 2016 to Dr. Michael P. Hengartner. The funder will have no bearing on the conduct, analysis, and interpretation of the data and the decision to submit a manuscript for publication. As stated above, all German-speaking patients starting psychotherapy at KGI in September 2015 and afterward will be included in the dataset. At present a total of *n* = 58 baseline and *n* = 6 follow-up measurements have been completed (effective October 2016). To achieve the projected sample size of *n* = 100 at 6-month follow-up, data collection will presumably continue until autumn 2017. Data analysis and manuscript draft will start immediately once the projected sample size has been reached. Study results are intended to be published in leading journals of clinical psychology, psychopathology, and psychotherapy.

## Anticipated Results

### Hypotheses and Planned Analyses

Some hypotheses were already roughly defined in winter 2015 and submitted to the KEK (see https://osf.io/ukbs5/). Here, we further detail those predictions and specify the statistical analyses that will be applied to test these hypotheses. The confirmatory analyses will be divided into two papers. Exploratory analyses will be published in additional papers, but these are not specified here.

First paper: the first confirmatory analysis will focus on the BFI. We will test the following hypotheses:

(1) BFI traits show high differential continuity over time (all *r* > 0.6). To test this prediction, we will compute bivariate Pearson correlations between trait scores at baseline and 6-month follow-up. We will provide both attenuated and disattenuated correlation coefficients corrected for scale unreliability.
(2) BFI traits demonstrate substantial mean-level continuity (all *d* < 0.5). More specifically, we predict that only neuroticism will demonstrate modest mean-level change (0.2 < *d* > 0.5), while the other four traits will remain almost unaltered over time (all *d* < 0.2). These predictions will be tested with *t*-tests for paired samples by examining the differences in standardized mean-level change between baseline and 6-month follow-up.
(3) Baseline BFI traits, specifically high neuroticism and low conscientiousness, substantially reduce (*d* > 0.5) the effectiveness of psychotherapy based on the BSI, GAF, and INK over the 6-month observation period. In order to test this prediction, we will compute GEE (121). These statistical models were introduced to fit regression analyses that account for within-subject correlation, which is an inherent part of longitudinal studies that rely on repeated outcome measures. Together with mixed models and random coefficient models, GEE represent the state of the art for longitudinal data analysis and are preferred to repeated measures ANOVA due to their superior psychometric properties (122, 123). The baseline BFI traits will be entered as the predictor variables and the repeated measures of the BSI, GAF, and INK successively as the outcome. The effect size of interest will be modeled as the interaction term between baseline personality and the time slope coefficient of the repeated outcomes, that is, the linear time trend for change in BSI, GAF, and INK. By simultaneously adjusting for the intercept, such a statistical modeling ensures that change in psychopathology over time is estimated independent of baseline impairment. These models will be conducted with and without adjustment for baseline diagnoses based on ICD-10.
(4) Mean-level change in BFI traits is only weakly, if at all, correlated with change in psychopathological impairment over time (all *r* < 0.3). To test this hypothesis, we will compute individual change scores over time for all repeated measures by subtracting the baseline score from the follow-up score. The change scores of the BFI traits will then be correlated with the change scores of the BSI, GAF, and INK using Pearson correlation.

Second paper: the second confirmatory analysis will focus on the IIP and IMI-C. We will test the following hypotheses:

(1) Self-reports and informant ratings of interpersonal personality problems are only modestly correlated (all *r* < 0.3). To test this hypothesis, we will subject the IIP (self-report) and the IMI-C (informant rating) to a bivariate Pearson correlation analysis.
(2) Both self-reported and informant-rated baseline personality problems according to the IIP and IMI-C substantially reduce (*d* > 0.5) the effectiveness of psychotherapy according to change in BSI, GAF, and INK from baseline to 6-month follow-up. As specified above with respect to the BFI, we will test these predictions by focusing on the interaction effect between baseline personality measures and the time slope coefficients of the psychopathology outcomes using a series of GEE analyses.
(3) Self-other agreement in personality profiles relates stronger to the outcome of psychotherapy than either self-report (IIP) or informant rating (IMI-C). To test this hypothesis, we will first compute the index of profile agreement (*I*_pa_) between IIP and IMI-C as recommended by McCrae (124) and then compare the slope-interaction effect of the *I*_pa_ with the multivariable effects obtained for the IIP and the IMI-C domains based on the results of GEE as specified above. The difference between two effect sizes will be considered statistically significant if the 95% confidence intervals of their standardized regression coefficients do not overlap.
(4) IIP and IMI-C have substantial incremental criterion validity (Δ*R*^2^ > 0.05) above and beyond each other in the prediction of the outcome of psychotherapy. This hypothesis will be tested by subjecting the change scores over time for BSI, GAF, and INK to a hierarchical linear regression analysis, where baseline IIP and IMI-C are entered in interchangeable hierarchical blocks as the predictor variables. In these models, the effect size of interest will be the amount of additional variance explained (Δ*R*^2^) accounted for by either IIP or IMI-C in the prediction of intraindividual change in BSI, GAF, and INK over time.

### Caveats and Potential Pitfalls

We have stressed that clinical trials in psychotherapy and psychopharmacology commonly have poor external validity, that is, the efficacy of treatments for mental disorders as estimated in the laboratory under controlled conditions using selective samples only poorly corresponds to real-world effectiveness in unselected naturalistic samples (47, 48). On the other hand, clinical trials have considerably higher internal validity than observational studies, which is why they are considered the gold standard for the estimation of causal effects. That holds particularly true when the main objective is to evaluate the efficacy of a given intervention. Observational studies are merely correlational, as interventions cannot be randomly assigned to participants. Therefore, they do not allow for strict causal conclusions, although sophisticated statistical techniques such as propensity score matching can approximate the internal validity of observational designs to that of randomized experiments (125). However, as we are mainly interested in the longitudinal covariance between personality and psychopathology and in the stability of personality, but not on the efficacy of psychotherapy as an intervention *per se*, that potential limitation is not at issue. That is, we are not interested in testing the efficacy of an intervention, but rather in evaluating possible mechanisms operating within that particular intervention. We will therefore not apply propensity score matching, as we do not compare psychotherapy users with untreated patients or other treatment modalities. A bias that needs to be addressed in such a study is the content overlap between personality and psychopathology, in particular that between neuroticism and depression (126), as that bias might artificially inflate the association between these constructs. To solve that problem, we will apply outcomes such as the GAF and the INK that have no content overlap with neuroticism. Moreover, we will also use alternative measures of personality such as the circumplex model of personality problems, which will be assessed based on both self-report and informant rating. Finally, though we consider the heterogeneity of this representative sample a particular strength, one might also argue that we compare apples to oranges. That argument holds particularly true with respect to interindividual differences in baseline impairment. As detailed above, we will resolve that issue by applying statistical models that estimate the change in psychopathology over time while holding baseline impairment constant. In order to additionally control for distinct baseline psychopathology, we will also include a patient’s primary diagnosis as a covariate.

## Summary and Conclusion

To increase the yield and validity of psychological research findings, direct replications of postulated associations are essential (51, 91). In order to strictly adhere to the tenets of confirmatory research (98), we prespecified not only our hypotheses but also the exact statistical analyses that we intend to conduct to test these *a priori* predictions. In line with an increasing demand for transparency and objectivity in scientific research (51), we published this research program publicly and made the original research protocol submitted to the local ethics committee that approved the study freely available (https://osf.io/ukbs5/). Most of the hypotheses outlined in this paper were prespecified in autumn 2015 and submitted to the responsible ethics committee in December 2015. Moreover, as of the writing of this paper (dating October 2016), only six follow-up assessments were completed, which precludes any prescreening of the data and hypothesizing in hindsight. By this means, we ensure that hypotheses and statistical approaches are specified before the data are collected and the results are known (98). Unfortunately, way too often hypotheses are specified after the results are known, and these *post hoc* analyses are then sold as confirmatory research based on *a priori* hypotheses (127), which substantially undermines the validity of research by inflating the false-positive rate (98). Therefore, we believe that ours is among the most stringent and transparent confirmatory research programs on the influence of personality on psychopathology and the outcome of psychotherapy under development to date. This study aims not only at replicating findings from selective RCT samples in a representative naturalistic sample; it will also address some major gaps in the scientific literature. These include for instance the stability of personality traits in a heterogeneous naturalistic sample of psychotherapy outpatients with diverse mental disorders others than major depression, the predictive validity of informant ratings of personality problems and how informant ratings of personality compare to corresponding self-reports. More than 20 years ago, Weisz and colleagues already noted that outcome studies conducted in the laboratory bear little validity for real-world clinical settings (128), an argument that was comprehensively reestablished approximately a decade later by Westen and colleagues (48). However, now, more than another decade later, almost nothing has changed. Findings on personality effects in psychotherapy studies are still largely based on highly selective samples under laboratory conditions, which is why we actually do not know whether these findings replicate in real-world clinical samples. We therefore believe that this confirmatory research program conducted in a representative naturalistic clinical setting can make a substantial contribution to the scientific literature on the pervasive impact of personality in the aetiopathology of mental disorders (4, 71) and corroborate (or, possibly, disconfirm) the important role of personality in the psychological treatment of these mental health problems (27, 74).

## Ethics Statement

This study was carried out in accordance with the recommendations of the Cantonal Ethics Committee (KEK) of Zurich with written informed consent from all subjects. All subjects gave written informed consent in accordance with the Declaration of Helsinki. The protocol was approved by the Cantonal Ethics Committee of Zurich.

## Author Contributions

MH designed the research program and drafted the manuscript. MY-A is the principal investigator at the study site and critically revised the manuscript. Both authors approved the final version of this manuscript.

## Conflict of Interest Statement

The authors declare that the research was conducted in the absence of any commercial or financial relationships that could be construed as a potential conflict of interest.
